# Perihilar cholangiocarcinoma masquerading as intrahepatic cholestasis of pregnancy: a case report and review of the literature

**DOI:** 10.3389/fmed.2024.1449865

**Published:** 2024-10-23

**Authors:** Chao Xiao, Cheng-Jian Cao, Xue Xiao, Qi-Jun Cheng

**Affiliations:** ^1^Department of Obstetrics and Gynecology, The First People’s Hospital of Zigong, Zigong, China; ^2^Department of Obstetrics and Gynecology, West China Second University Hospital, Sichuan University, Chengdu, China; ^3^Zigong Academy of Medical Sciences, Zigong, China

**Keywords:** perihilar cholangiocarcinoma, pruritus, jaundice, intrahepatic cholestasis of pregnancy, case report

## Abstract

Cholangiocarcinoma (CCA) is a tumor that arises from the epithelium of the intrahepatic bile ducts. It is rarely diagnosed in individuals under 40 years of age and has a very aggressive nature: 95% of patients die within 5 years. We present the first detailed report of a case of perihilar CCA (pCCA) presenting during pregnancy masquerading as intrahepatic cholestasis of pregnancy (ICP). First, the patient exhibited typical pruritus, particularly in her limbs; second, a raised biomarker of total bile acid (TBA) was noted; third, the onset occurred in the second trimester, aligning with the epidemiological profile; and finally, importantly, there was no mass detected in her liver. First-line drugs were given to treat ICP, but they failed, and ultimately, the condition was identified as pCCA. Following an inadequate excision, traditional Chinese medicine was administered. After 26 months, she succumbed to cachexia. As gestational symptoms are sometimes associated with pregnancy-related disorders, pCCA in pregnant women is frequently misdiagnosed. Symptoms such as jaundice, pruritus, and dilated bile ducts in pregnant women may indicate pCCA. In addition, the appropriate treatment for pCCA in pregnant women may be surgery or chemotherapy; if surgery is not an option, chemotherapy may also help extend the gestational week. Our work is important and can educate on the diagnosis and treatment of pregnancy-related diseases, such as ICP and pCCA.

## 1 Introduction

The incidence of malignant tumors in pregnant women ranges from 0.05 to 0.10% (1) and is associated with advanced maternal age and obesity during pregnancy (2). Gestational neoplasms are defined as malignant tumors that emerge within a 12-month window following pregnancy, up to 3 months before the scheduled termination of a pregnancy, or up to 9 months before delivery. Due to their atypical clinical manifestations, these tumors are frequently mistaken for normal physiological changes associated with pregnancy, significantly complicating the diagnostic process. Consequently, the clinical diagnosis of such tumors often occurs at an advanced stage (3). The median survival time for cholangiocarcinoma (CCA) is only 3–6 months, with an incidence rate of 0.58 per 100,000 individuals (4). CCA is traditionally categorized into intrahepatic CCA (iCCA), perihilar CCA (pCCA), and distal CCA (dCCA). A case of an itchy pregnant woman diagnosed with CCA has been documented (5), featuring an unusually large, central malignant liver tumor. Here, we present the first detailed report of a pCCA case characterized primarily by pruritus typical of intrahepatic cholestasis of pregnancy (ICP)—a prevalent pregnancy-related complication. Obstetricians may more frequently misdiagnose pCCA as ICP in practice. In addition, we conducted a comprehensive review of the literature to explore the diagnosis, treatment, and prognosis of CCA in the context of pregnancy.

## 2 Case

A 33-year-old woman in her fourth pregnancy, with one living 6-year-old daughter, presented to our department at a gestational week (GW) of 21^4/7^, complaining of cutaneous pruritus for 13 days. Her height was 159 cm, her weight was 71.5 kg, and her body mass index (BMI) was 27.3. She was receiving antenatal care at a local hospital. Routine tests were within the normal range. She had no previous medical history of drug abuse, liver, immunologic, or viral problems, and no history of ICP in her previous pregnancy. Her mental status, appetite, sleep, and weight were normal, with no other obvious symptoms except for pruritus. Her liver was not palpable below the costal margin. Her scleral icterus appeared at 21 weeks of gestation. The height of the uterus was appropriate for her GW.

Moreover, 4 days before her admission to our Municipal Health Care Hospital (11 December 2020), she had itchy skin all over her body, particularly on her limbs. Liver function tests revealed that total bile acid (TBA) was 214.6 μmol/L (range: 0–10 μmol/L), alanine aminotransferase (ALT) was 201.6 U/L (range: 9–50 U/L), and aspartate aminotransferase (AST) was 117.7 U/L (range: 18–40 U/L) (details in Table 1). The obstetrician prescribed first-line treatment with glutathione and ursodeoxycholic acid (UDCA) to treat ICP, which was diagnosed based on the clinical data and epidemiological characteristics, as it is a high-incidence disease in Sichuan province, China. Other tests were arranged, including liver immunology and viral serology, both of which subsequently yielded negative results. Ultrasound showed intrahepatic bile duct dilatation without further examination. After 1 week of medication, her liver function was retested. The results showed that TBA was 254.8 μmol/L, ALT was 106.9 U/L, and AST was 58.4 U/L. However, her pruritus worsened, and there was no decrease in her TBA levels, leading to her transfer to our department on 22 December 2020.

**Table 1:** Liver function and treatment details.

Test time	ALT (9–50)	AST (18–40)	TBA (0–10)	TBIL (0–23)	DBIL (0–10)	IBIL (5–18)	ALB (40–55)	Treatment
24.12.2020	139	75	261.7	22.9	19	3.9	34.8	UDCA 250 μg Q8h glutathione 1.2 g Qd
28.12.2020	108	51	280	26.9	22.6	4.3	32.1	UDCA 250 μg Q6h glutathione 1.2 g Qd
31.12.2020	80	39	301.6	40.8	34.5	6.3	30.4	Abortion
05.01.2021	42	32	279.5	59	45.5	13.6	28.7	UDCA 250 μg Q6h
07.01.2021	30	24	208.2	68.3	55.9	12.4	25.9	Discharge
19.02.2021	31	25	69.1	21.8	14.4	7.4	31.8	Adjuvant stage

The unit of measurement of TBA, TBIL, DBIL, and IBIL is μmol/L; the unit of measurement of ALT and AST is U/L; and the unit of measurement of ALB is g/L.

We reviewed her medical history for this pregnancy and retested her liver and thyroid functions 2 days later. The patient’s vital signs were recorded as follows: temperature at 36.3°C, heart rate at 86 beats per min, and blood pressure at 113/56 mmHg. Her liver enzymes were elevated, with ALT at 139 U/L and AST at 75 U/L. Her TBA level was 261.7 μmol/L, direct bilirubin was 19.0 μmol/L, alkaline phosphatase was 397 U/L, uric acid was 462 μmol/L, and thyroid-stimulating hormone was 0.798 mIU/L (Table 1 for details). We prescribed her UDCA 250 μg every 8 h, levothyroxine sodium tablets at a dose of 25 mcg daily taken orally, and intravenous glutathione at a dose of 1.2 g daily. Her platelet count fluctuated between 217 and 279 × 10^9/L. Despite treatment, she experienced uncontrollable pruritus and jaundice, which made it difficult for her to sleep. Her TBA levels continued to rise even while she was on UDCA. After a thorough discussion with her family, she decided to terminate the pregnancy. Consequently, we performed a double-balloon induction of labor on 31 December 2020. Oxytocin and misoprostol were administered for the next 4 days with no effect. On 5 January 2021, ethacridine lactate amniocentesis was performed to induce labor. On 7 January 2021, the pregnancy tissue was delivered.

**Figure 1:**
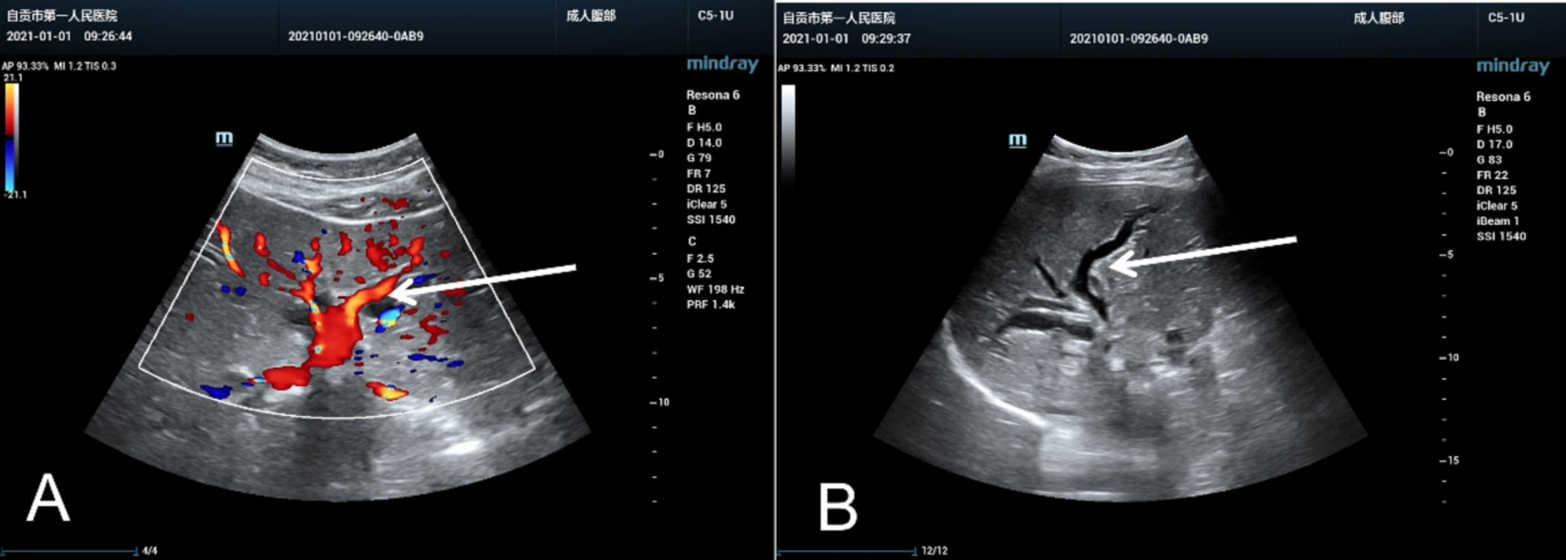
The ultrasound images of the liver resembled a spider or an evil spirit walking in the hepatic hilum of the pregnant woman. The oblique diameter of the right liver was approximately 14.5 cm. There were echoless areas in the liver, and the larger one was approximately 2.2×1.5 cm. The liver capsule was not smooth, the internal diameter of the main portal vein was approximately 1.0 cm, and dilatation of the bile duct was evident without mass effect in the hepatic hilar. **(A)** Color Doppler ultrasound image and **(B)** Two-dimensional ultrasound image, marked with a white arrow.

**Figure 2:**
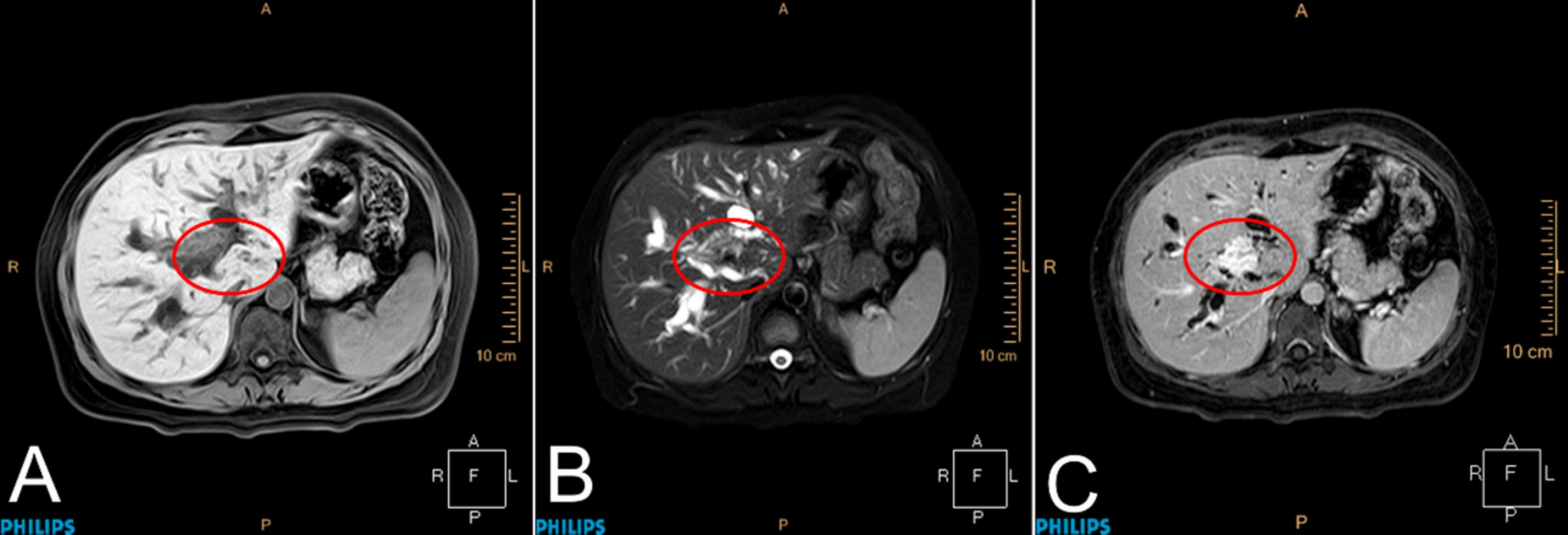
Magnetic resonance imaging of the liver. Patchy abnormal signals were observed at the confluence of the left and right hepatic ducts in the hilar region of the liver, with a maximum cross-sectional area of approximately 2.0 × 1.4 cm. Enhanced scanning showed gradual and uniform enhancement. The lesion was distributed along the bile duct, and the distal bile duct wall was slightly thickened and enhanced. The proximal bile ducts were significantly dilated. The lesion was adjacent to the main left and right branches of the portal vein. Portal lymph nodes were visible. **(A)** T1WI, **(B)** T2WI, and **(C)** DCE-T1, marked with a red circle.

Reexamination of the liver by ultrasound on 1 January 2021 showed dilatation of the intrahepatic bile duct with a disorder of echo in the first hepatic portal area. The sonographer suggested pCCA with the presence of a spider sign (Figure 1). An enhanced MRI was arranged on 6 January 2021, which showed a hepatic portal nodular abnormal signal with bile duct obstruction (Figure 2). The diagnosis of pCCA was considered after a multidisciplinary consultation, and we advised her to undergo surgical treatment at the Hepatic Center (Western China Hospital).

**Figure 3:**
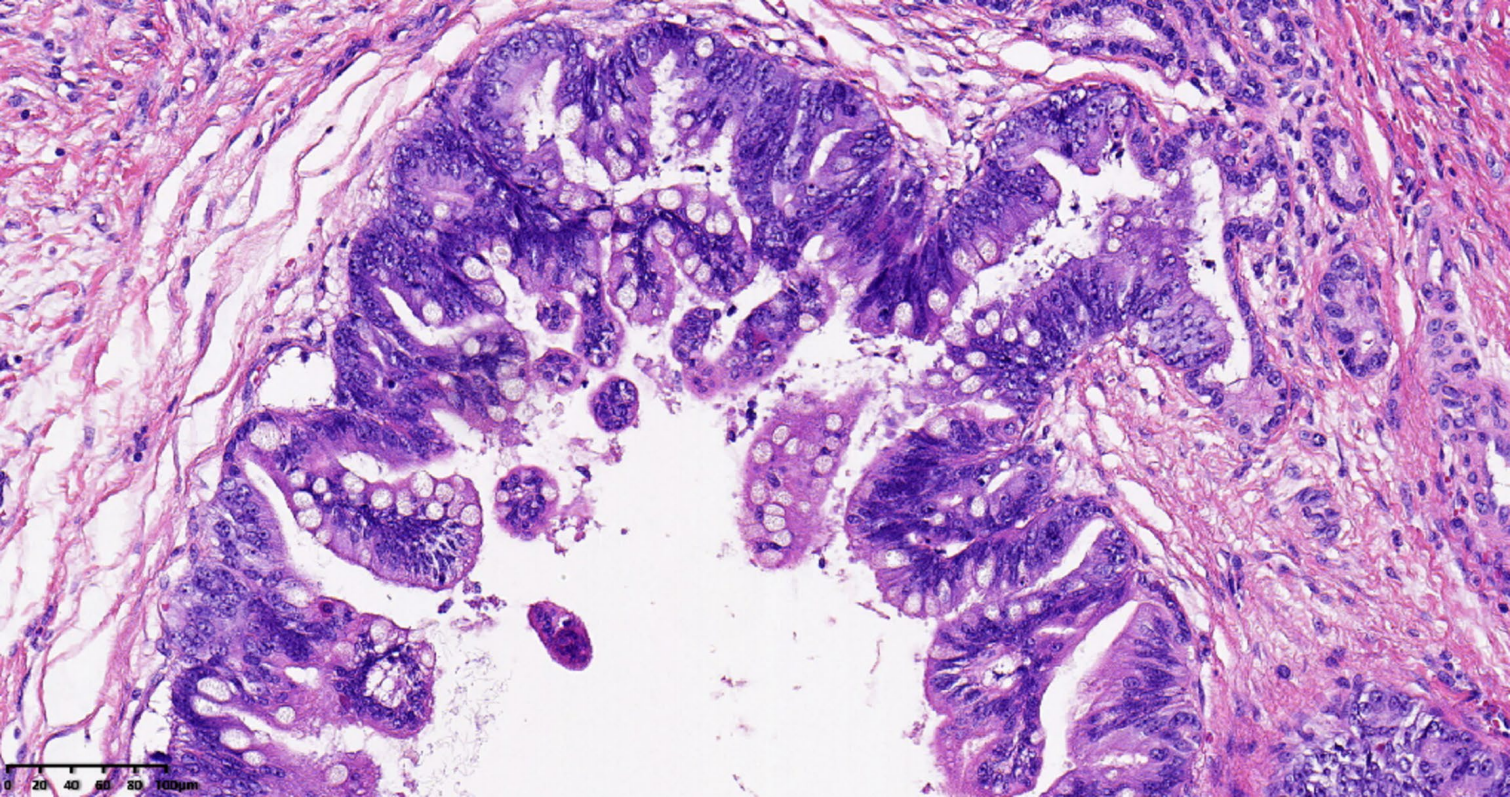
HE staining of the tumor shows invasive cholangiocarcinoma in hematoxylin and eosin staining.

She was admitted to the biliary surgery department of the West China Hospital of Sichuan University and underwent surgery on 18 January 2021. The surgical findings revealed yellowish ascites surrounding a dark brown liver that did not have nodular masses on its surface. Lymph nodes were palpable behind the hepatoduodenal ligament, around the cystic triangle and cystic duct, and next to the common hepatic artery. Unfortunately, intraoperative pathological examination showed that the carcinoma had metastasized to the common bile duct section, the left and right intrahepatic bile ducts, the cystic duct, and the wall of the common hepatic duct. Radical resection was not possible; instead, a median hepatectomy, hilar choledochoplasty, and cholecystojejunostomy were performed. She was transferred back to our hospital’s hepatobiliary surgery department for further treatment on 29 January 2021. The final diagnosis was pCCA (classified as Bismuth stage IV and according to the American Joint Committee on Cancer (AJCC) classification as T2N1M0, stage IIIB) (Figure 3). After 3 months following the unsatisfactory resection, her mental status, appetite, and sleep had normalized, and she no longer experienced pruritus. However, she had lost 10 kg of body weight. She visited her surgeon on 1 April 2021. The surgeon advised her to consider traditional Chinese medicine but did not recommend chemotherapy, citing a lack of sensitivity to any drugs. She suffered from constant abdominal pain and experienced weight loss due to poor appetite. After 26 months, she succumbed to cachexia on 14 March 2023.

## 3 Review of the literature

The PubMed, Wanfang, and Embase databases were searched, and only 16 cases in 15 reports of CCA during pregnancy from 1975 to 2021 were identified (5–18), as shown in Supplementary Table 1. The characteristics of these CAA patients were identified after a systemic review. The ages of these 16 women ranged from 22 to 39 years. Moreover, 3 out of the 16 women were primiparas (9, 10, 14), while the most prolific woman had been pregnant nine times (6). Only two cases (12, 18) had full-term deliveries, while the other cases were either abortions or premature deliveries, with the earliest abortion at 18 GWs. Seven cases underwent cesarean section due to pain, acute fatty liver of pregnancy (AFLP), HELLP (H = hemolysis, EL = elevated liver enzymes, and LP = low platelets) syndrome, and pre-eclampsia. The remaining cases were vaginal deliveries. Four newborns died, while the others survived. The onset of symptoms ranged from 16 GWs to 2 weeks postpartum. The most common symptoms included right upper quadrant pain, emesis, fever, nausea, fatigue, anorexia, pruritus, and weight loss. Only one case presented with spinal cord compression (13). Abnormal examination findings included palpable liver and jaundice. Imaging findings showed “intrahepatic biliary dilatation“and “mass lesions in the liver.” Seven cases were diagnosed by biopsy. One patient died of meningitis during pregnancy, and an autopsy revealed incidental iCCA, while another case involved a postpartum woman who had a positive pregnancy test and was found to have metastatic cancer (6). Liver enzymes (AST and ALT) were normal or slightly elevated in the laboratory tests of the reported cases, except for the case that we reported, which showed levels twice the upper limit range. TBIL levels were high in nine cases (range: 0.5–22.9 mg/dL), with one case reaching 271.94 mol/L (11). AFP values of 9.21–29.6 IU/mL or 246.2–1800 ng/mL were observed in various units. Hepatitis B surface antigen and hepatitis C antibody were negative in seven cases. HELLP (10), AFLP (16), and ICP (5), along with the case we presented, all mimicked the diagnosis of CCA. Only five cases were treated surgically, two of which required hepatectomy. The majority of the patients chose chemotherapy, with gemcitabine being the most widely used drug (14–16). The prognosis was generally dismal; eight of the women died soon after the diagnosis. The longest period of follow-up was 17 months (15). Without undergoing radical resection or aggressive therapy, one patient died of cachexia 26 months later.

## 4 Discussion

CCA accounts for approximately 10% of all liver cancer cases and 3% of all gastrointestinal malignancies (19). In 2020, primary liver cancer was reported to be the sixth leading cause of new cases worldwide, with approximately 906,000 cases, and the third leading cause of cancer-related deaths, with approximately 830,000 cases (20). CCA is associated with intrahepatic cholangitis, liver fluke, non-alcoholic fatty liver disease, obesity, diabetes, and hepatitis B virus infection (21, 22). These risk factors contribute to chronic inflammation of the bile ducts and cholestasis, altering the microenvironment of the bile ducts and increasing the risk of cholangiocarcinoma (23). Oncogenes (RAS, ERBB2, BRAF, EGFR, PIK3CA, and CTNNB1) and tumor suppressor genes (p53, Smad4, and CDKN2A) are also involved in the development of intrahepatic cholangiocarcinoma (24–26). In the case we reported, the only risk factor was obesity, with a BMI of 27.

The clinical manifestations of gestational tumors often mimic pregnancy-related symptoms, leading to a late diagnosis of the tumor (27). Fatigue, fever, night sweats, abdominal pain, and jaundice are the most common symptoms of CCA, which is consistent with previous reports (28). However, the case we presented in this study was more elusive and lacked the typical liver lesions associated with occupational exposure. We described in detail the first case of pruritus that led to a clinical misdiagnosis of ICP. The presence of a hilar lesion upon reexamination is only considered if bile acid levels do not decrease and bilirubin levels rise throughout treatment, ultimately leading to a diagnosis of pCCA. When clinical signs of probable cancer are discovered during pregnancy, including increased markers, jaundice, and a dilated bile duct, they should be thoroughly evaluated. Ultrasound scanning does not involve ionizing radiation, making it the preferred examination method for pregnant women (29). MRI may also be used as a complementary examination, but contrast enhancement agents must be avoided at all costs. Gadolinium is associated with an increased risk of fetal stillbirth and neonatal death, as well as inflammatory, rheumatic, and invasive skin lesions in children (30). Endoscopy of the digestive tract during pregnancy is considered a safe procedure for both the mother and the fetus. Elevated CA15-3 levels during pregnancy are associated with ICP (31), and other tumor markers are not specific during pregnancy, so they should not be used as the basis for diagnosing tumors during pregnancy (32–34).

In the majority of cases, a combination of surgery, chemotherapy, and radiation therapy can be used during pregnancy. Although CCA patients are often diagnosed at a later stage, with a postoperative recurrence rate of up to 64%, surgical resection is the only curative treatment option (35), and can be performed by a multidisciplinary team of experienced surgeons, anesthesiologists, and obstetricians. One pregnant woman with CCA underwent surgical resection at 30 weeks of gestation, and the pregnancy was terminated by cesarean section at 38 weeks of gestation (18). Seven out of the nine women who received chemotherapy had fetuses older than 30 weeks old. Trastuzumab reportedly provided complete relief to a pregnant patient suffering from liver metastasis caused by a breast tumor, and the development of the fetus proceeded normally (36). In our reviewed cases, capecitabine, gemcitabine, and cisplatin were the most commonly used chemotherapeutic agents.

## 5 Conclusion

We have presented the first detailed report of a pCCA case during pregnancy masquerading as ICP with the most classic manifestations: first, she exhibited typical pruritus, particularly in her limbs; second, there was a raised biomarker of total bile acid (TBA); third, the onset occurred in the second trimester, aligning with the epidemiological profile; and finally, and importantly, there was no mass detected in her liver. It is both meaningful and educational for medical professionals, especially those who work in areas with a high incidence of ICP, to approach the diagnosis and treatment of this pregnancy-related ICP without relying on inertial thinking. Symptoms such as jaundice, pruritus, and dilated bile ducts may be indicators of CCA. Furthermore, the appropriate treatment for CCA during pregnancy may be surgery or chemotherapy. If surgery is not an option, chemotherapy may also help extend the gestational week.

## Data Availability

The original contributions presented in the study are included in the article/Supplementary material, further inquiries can be directed to the corresponding authors.

## Supplementary material

SUPPLEMENTARY TABLE 1 Cholangiocarcinoma diagnosed in pregnancy.
